# A Suggested Strategy to Integrate an Elective on Clinical Nutrition with Culinary Medicine

**DOI:** 10.1007/s40670-021-01346-3

**Published:** 2021-07-06

**Authors:** Lindsey K. Leggett, Kareem Ahmed, Cheryl Vanier, Amina Sadik

**Affiliations:** College of Osteopathic Medicine, Touro University Nevada, Henderson, NV USA

**Keywords:** Clinical nutrition, Culinary medicine, Preventive health education, Undergraduate medical education

## Abstract

Time allocated to nutrition education in the medical school curriculum stands in contrast to high mortality rates attributable to poor diet in patients. Counseling patients on nutrition-related diseases is a critical skill for physicians, particularly those entering primary care. The crowded medical school curriculum has made adding hours of nutrition instruction difficult. This study evaluates the attitudes of undergraduate medical students at a single institution regarding the need for and relevance of nutrition education and reports on organization of and students’ responses to a short experimental elective. Student attitudes regarding nutrition in medicine and a proposed nutrition elective were surveyed. Results helped formulate a short experimental elective. A two-session experimental course was completed, after which the participants were surveyed. Students agreed or strongly agreed with statements regarding the importance of nutrition in clinical practice. Greater than 60% of students surveyed in each class were interested in the proposed elective. All participants found the elective with culinary medicine sessions at least moderately useful to their needs as future healthcare providers. The majority of participants (more than 93%) reported being likely to both take and recommend the elective should it be offered in the future. Medical students consider nutrition an essential aspect of a patient encounter, but do not feel prepared to counsel future patients on dietary changes for management and/or prevention of nutrition linked diseases. There is strong student support for creating an elective in clinical nutrition with culinary medicine sessions to address the gap in their education and improve their confidence.

**Supplementary Information:**

The online version contains supplementary material available at 10.1007/s40670-021-01346-3.

## Introduction

Seven of the top ten causes of death globally are attributable to non-communicable causes, including cardiovascular disease (CVD), stroke, chronic obstructive pulmonary disease, cancers attributable to smoking, dementia-producing diseases, diabetes mellitus, and kidney disease [1]. Non-communicable diseases are particularly prevalent in high-income countries, where most communicable diseases have been successfully controlled. For example, in the USA, heart disease, stroke, and diabetes are in the top seven causes of death [2].

Treatment and prevention of non-communicable diseases is therefore an important topic for health care professionals, yet the most effective interventions are often overlooked in medical education. The CDC estimates that the elimination of three key risk factors: poor nutrition, inactivity, and smoking, would prevent 80% of heart disease, stroke, and type 2 diabetes. Of the three, nutrition is the most critical component to preventing premature CVD deaths [3, 4] as well as helping manage or prevent other non-communicable diseases. For example, heart disease, stroke, and diabetes are often preceded by elevated blood pressure, raised blood glucose and cholesterol levels, and excess body weight, all of which may be addressed by a healthier diet. Diet has been identified as the single most significant risk factor for disability and premature death in the USA [5].

Coverage of nutrition and its influence on health issues in the medical education curriculum is not in alignment with the prevalence and seriousness of the diseases stemming from a poor diet. There is an abundance of research indicating nutrition is a key component in preventing or managing important and common diseases, and the poor coverage within the medical school curriculum has been recognized for over 30 years. The National Academy of Science recommended in 1985 that medical schools include a minimum of 25 hours dedicated to nutrition [5]. However, in 2015, a study which included 121 allopathic medical schools in the USA found that only 29% met or exceeded the recommended hours of instruction [6]. A separate study of 26 osteopathic medical schools revealed that only 15% met the recommended hours of nutrition instruction [7].

The sub-optimal level of nutrition education is particularly surprising in osteopathic medical schools. Osteopathic medicine was founded by A.T. Still, who asserted that environmental, cultural, social, mental, and behavioral factors should be addressed by any management plan produced by a health care provider [8], indicating a whole-person, patient-centered philosophy for treating disease. Schools that produce osteopathic doctors (DOs) are primarily responsible for training primary care providers. In 2019, 56.5% of osteopathic physicians identified their specialty as primary care [9]. By virtue of their guiding philosophy and the primary care focus of their students, osteopathic medical education should be at the forefront of preventative medicine [7].

It is important that primary care providers (PCP) be equipped to recognize, coordinate, and manage diet-related problems and health conditions because they are the members of the health care team who make initial contact with a patient and decide on who else should be involved in the patient’s care [10]. Although it is true that registered dieticians and nurse practitioners may be the members of the health care team who have proven effectiveness for patients in the area of nutrition counseling [10], the PCP must first recognize that nutrition is a component of a patient’s problem and involve the necessary personnel. If a PCP has knowledge of nutrition, he or she is more likely to think about nutrition counseling as a component of care to prevent, reverse, or improve outcomes in non-communicable disease. If a PCP personally practices the tenets of good nutrition, even greater gains may be realized.

Poor coverage of nutrition in the medical school curriculum prevents physicians from taking their rightful place as nutrition advisors and lifestyle role models to prevent or manage nutrition related diseases. A majority of patients (61%) consider doctors to be “very credible” sources of nutritional information [11], and some of the credibility is based on perception of the physician as a role model. For example, patients who watched a video of physicians disclosing their own healthy habits regarding both exercise and diet found the physicians to be significantly more believable and motivating when counseling individuals on nutrition and a healthy lifestyle [12]. There is ample evidence that physicians who practice healthy nutrition habits are more likely to counsel their patients on healthy habits [13, 14]. The current reality is that primary care providers report difficulty in counseling patients about behaviors that they themselves struggle with and do not practice [15]. In a recent study, only 14% of resident physicians believed themselves adequately trained to provide nutritional counseling [11].

Therefore, there is a significant need for medical schools to offer a strong nutrition education curriculum with an emphasis on managing special diets, not only for patients but also for future physicians. This curriculum should also include guidance on how to counsel patients in a sensitive and professional manner as improving nutrition can require major lifestyle changes, and counseling is more effective when the person recommending a change exemplifies the desired behavior in his or her own life. Most nutrition education takes place during the preclinical years, with very little instruction received during clinical training [6], so it is during undergraduate medical education (UME) that the problem should be addressed. The UME curriculum is crowded with requirements associated with accreditation and standardized examinations, so solving the problem associated with delivering an adequate nutrition education calls for a creative approach, such as offering a nutrition education elective with an active learning component.

Nutrition education is ideally suited for experiential learning because the purpose of nutrition education is not only to provide accurate information about the updated practices in the subject matter, but also to influence future physicians to adopt healthier nutritional behaviors. Mauriello and Artz reported on the burden of chronic diseases on health care costs and the need for focusing more of health care on lifestyle and nutrition to reduce these costs [16]. They indicate that training future physicians using a hands-on approach called “culinary medicine” is the ideal method to alleviate the burdens associated with nutrition-related chronic disease [16]. La Puma defined culinary medicine as “a new evidence-based field in medicine that blends the art of food and cooking with the science of medicine” [17]. He adds that “culinary medicine is aimed at helping people reach good personal medical decisions about accessing and eating high-quality meals that help prevent and treat disease and restore well-being” [17]. It gives participants the opportunity to learn not only the pathophysiology behind nutrition-linked diseases and which foods to use for specific conditions, but also teaches how to transform ingredients into tasty meals. The goal of culinary medicine is to impart knowledge that physicians can then use to improve their own diets in addition to advising patients in practical ways. Medical students trained in culinary medicine, as opposed to traditional education, report an improved fruit and vegetable diet and better attitudes and competencies in the area of nutrition [17].

The purpose of this descriptive study was to explore an alternative way to provide nutrition education without adding hours to the formal curriculum by (1) surveying student perceptions regarding current nutrition education, (2) surveying student interest in attending a nutrition elective, (3) selecting how the elective could best be delivered, and finally (4) running and assessing participants’ reactions to a short experimental version of the elective. The overarching goal was to provide future physicians with relevant and actionable nutrition education through a focused clinical nutrition elective with a culinary medicine component.

## Materials and Methods

### Needs Assessment Survey

Two surveys, one designed for first- and second-year medical students (Table 1 in Appendix) and the other for third- and fourth-year medical students (Table 2 in Appendix), were derived from validated questionnaires previously published by Walsh et al. and Hardman et al. [18, 19]. Both surveys gathered information on demographics, opinions about the current nutrition instruction, and students’ interest in a clinical nutrition elective supplemented by culinary medicine workshops and the delivery preference for such an elective. The first- and second-year cohort survey additionally assessed the relevance of nutrition in disease prevention. Because they are already in clinical rotations where they see patients, the third- and fourth-year cohort survey additionally focused on their feelings of preparedness to counsel patients regarding lifestyle changes and the perceived usefulness of the proposed elective. A 5-point Likert scale (strongly disagree, disagree, neutral, agree, and strongly agree) was used to indicate agreement with the survey statements.

The survey was sent to all medical students at Touro University Nevada College of Osteopathic Medicine (TUNCOM). Because of a recent curriculum change, the third-year and fourth-year students who were surveyed had received four hours of nutrition education, whereas the first-year and second-year students had received 0 hours of nutrition education at the time the survey took place. The recruitment of participants was done via email with a link to complete the survey on SurveyMonkey. Students consented to participate by initiating the survey. Responses were collected and de-identified by a third party. A descriptive analysis was performed using Excel and R Software.

### Experimental Culinary Medicine Workshops

Utilizing the results from the needs assessment surveys, a short experimental version of the elective course was organized to determine the feasibility of the proposed elective. The experimental course consisted of a didactic unit and two culinary medicine workshops. The didactic portion was to be completed before the second culinary medicine session and was composed of (1) a PowerPoint on coronary artery disease causes and nutritional preventive measures, (2) a document about patient counseling prepared by a certified nutritionist, and (3) an assessment in the form of two clinical cases with essay answers.

The first culinary medicine session was dedicated to learning food safety and knife skills. The second session focused on preparing food for a patient who is on a sodium and fat restricted diet. Discussion about the content of the didactic assessment as well as the workshops was held at the end of the second workshop session while students were eating the dinner they prepared.

A call for participation was sent to all students of TUNCOM. The first four responders from each year were chosen to complete the short experimental course, for a total of 16 students which was dictated by the capacity of teaching kitchen where the workshop took place. Participants signed a consent form before the workshop. A local culinary school volunteered its facilities and resources to complete the culinary medicine sessions. One of the teaching chefs conducted the sessions. The ingredients to prepare the meal and the chef’s honorarium were covered by the IAMSE ScholarRx Student Grant.

### Post Workshop Survey

A post workshop survey was created to gather feedback on the short experimental culinary medicine elective (Table 3 in Appendix). The survey questions assessed participants’ confidence in preparing meals, satisfaction with the organization and the process of the short experimental course, the likelihood of participating or recommending the proposed elective to a friend and gave an opportunity for suggestions for improvement. The survey was administered using Qualtrics. The majority of participants completed the survey (15 of 16). The reports were downloaded, and data was analyzed using descriptive statistics, since the study did not include any testable hypotheses.

This study was determined exempt by the Touro University Nevada Institutional Review Board (#TUNIRB000028).

## Results

The survey response rate was 66% for first-year students and 22%, 36%, and 13% for 2nd, 3rd, and 4th year students (Table 1). Demographics of respondents were similar to those of the entire class of students. A majority of students did not have nutrition training prior to medical school but indicated that someone close to them has a nutrition-linked problem that requires some knowledge of nutrition. The most common intended specialties for students included primary care (family medicine, internal medicine, Obstetrics, and Gynecology), emergency medicine, surgery, or anesthesiology.

**Table 1:** Demographic information for survey respondents

		1st year	2nd year	3rd year	4th year
Sample size		124	29	49	17
Gender	%Male	55%	55%	54%	59%
Age	18–24	38%	17%	0%	0%
	25–34	62%	79%	100%	94%
	35–44	0%	3%	0%	6%
Race/Ethnicity	American Indian/Alaska Native	0%	0%	0%	0%
	Asian	42%	31%	31%	31%
	Black/African-American	1%	0%	2%	0%
	White	39%	38%	55%	56%
	> 1 of the above	7%	17%	4%	6%
	Other	8%	10%	8%	6%
	Hispanic or Latino*	3%	3%	4%	12%
Did you have any nutrition training prior to medical school?	No	57%	62%	63%	76%
	Yes, college coursework	33%	34%	24%	18%
	Yes, RD		0%	0%	0%
	Yes, PhD		0%	0%	0%
	Yes, Physical trainer		0%	6%	0%
	Yes, Other	11%	3%	6%	6%
Do you or any of your close family members or friends have any nutrition-linked medical problems that would require a greater than average knowledge of nutrition (i.e., diabetes, BMI)?	Yes	75%	69%	63%	59%
Which of the following represents where your GPA stands thus far?	< 2.0	0%	0%	0%	0%
	2.0–2.5	6%	0%	12%	0%
	2.5–3.0	21%	17%	18%	18%
	3.0–3.5	53%	72%	49%	59%
	3.5–4.0	20%	10%	20%	24%
What is your intended specialty?	Anesthesiology	2%	10%	9%	6%
	Dermatology	0%	3%	0%	0%
	Emergency Medicine	13%	14%	4%	6%
	Family Medicine	14%	10%	21%	44%
	Internal Medicine	17%	10%	15%	6%
	Neurology	2%	3%	0%	0%
	Obstetrics and Gynecology	4%	3%	17%	6%
	Ophthalmology	0%	0%	0%	0%
	Pathology	0%	3%	2%	0%
	Psychiatry	2%	7%	9%	19%
	Radiology	1%	0%	2%	0%
	Surgery	12%	7%	4%	6%
	Undecided	32%	28%	17%	6%

*For 1st and 2nd year respondents, “Hispanic or Latino” were included in a combined race/ethnicity category, whereas for 3rd and 4th year respondents, “Hispanic or Latino” was offered as an “Ethnicity” question, separate from “Race”

**Fig. 1:**
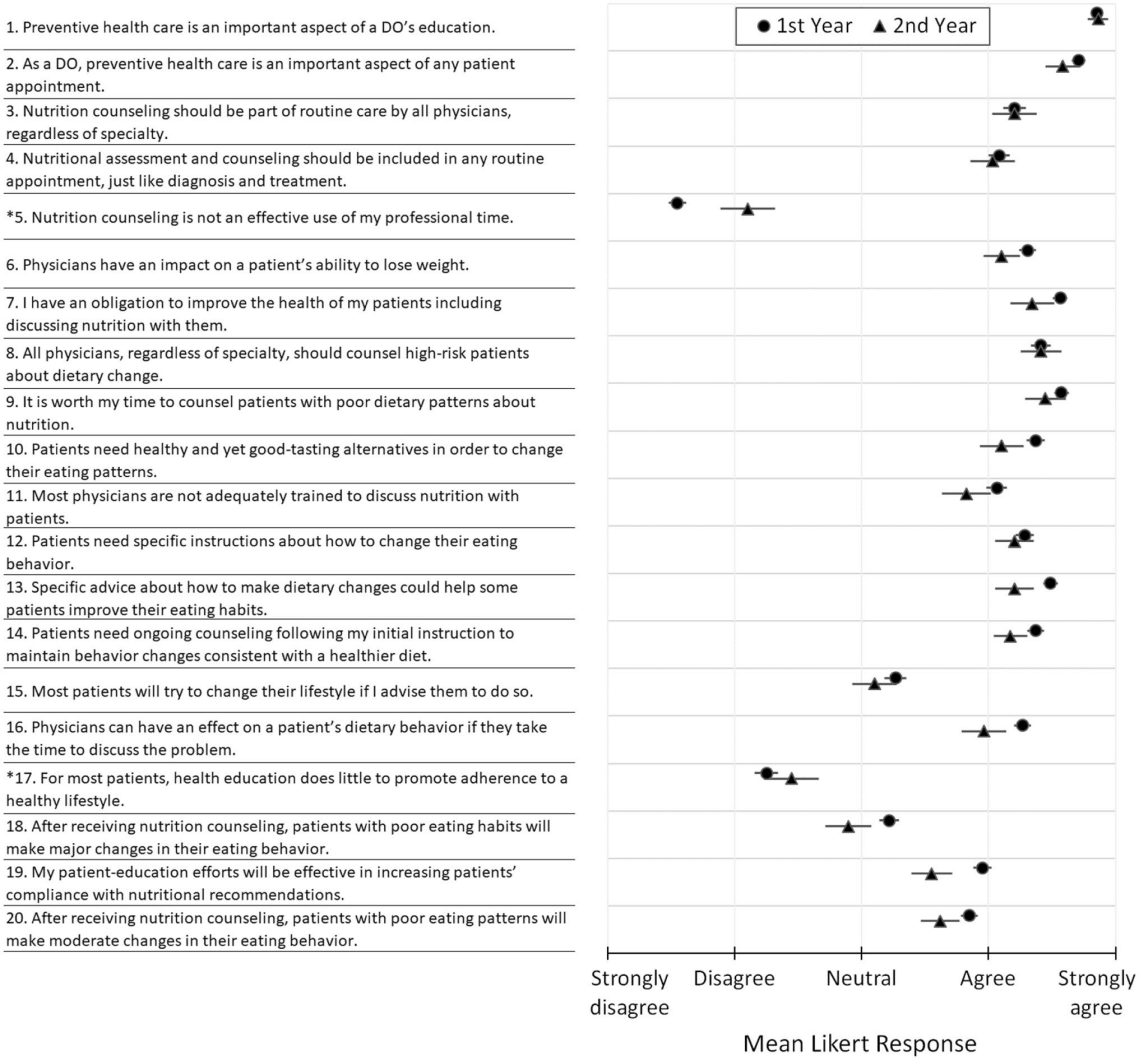
Mean response (SE) to questions regarding physician responsibilities and impacts on patient nutrition.“Asterisks” indicate questions which are worded to suggest a negative view of nutrition education, whereas all others use positive wording

First- and second-year students strongly agreed that preventive health care is an important aspect of a physician’s education and should be an important part of patient interactions (Fig. 1). They also agreed or strongly agreed that nutrition counseling was an important component of patient care and that physicians are not adequately trained in nutrition. Finally, first- and second-year students believed it is important to counsel high risk patients about dietary changes and should be given specific instructions on how to improve their eating habits. Students tended to be less confident in a physician’s ability to get a patient to change his or her lifestyle, and first-year students tended to have a more positive attitude regarding potential physician impacts compared to second-year students.

**Fig. 2:**
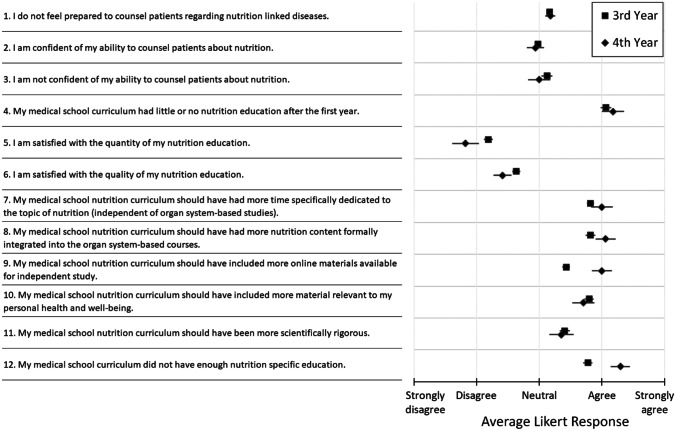
Average response (SE) to questions regarding 3rd and 4th year students’ perception of their nutrition education

Third- and fourth-year students were on average neutral regarding their own nutrition education, although they indicated that they should have had more time devoted to nutrition specific education (Fig. 2). Third- and fourth-year students were neutral on their ability to counsel patients on nutrition. Fourth-year students were less satisfied with their nutrition education, on average, compared to third-year students.

**Fig. 3:**
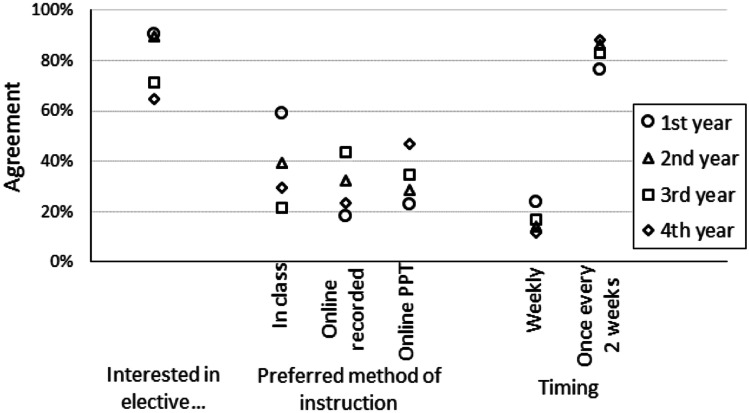
Interest in elective nutrition course and details of delivery across medical student levels

More than 60% of students in all 4 years of medical school were interested in an elective nutrition course, although interest dropped by approximately 20% between the first 2 years and the second 2 years (Fig. 3). The most popular method of instruction depended on the year of the student. First-year students preferred in-class instruction. The other years preferred online instruction with a similar split between online recorded classes or online PowerPoints. All levels of students preferred a once every two weeks format by a wide margin.

The feedback from the limited number of students who attended the short experimental sessions was positive overall. All but one of the 15 students reported that the sessions met their expectations, the format was suitable, and that the list of topics to be covered by the elective course was complete. Almost all participants gave positive ratings to the workshop for improving cooking skills and the quality of the sessions. Most importantly, participants were willing to take the elective if offered, and they were overall very likely to recommend the elective to other students. Participants were less certain about the applicability of the elective towards counseling future patients (Table 2).

**Table 2:** Post-workshop rating survey. The numbers of respondents are reported for each Likert scale rating, and the mean and standard error (SE) are reported for each question. Yes or no answers are reported as the percent of positive responses

		Likert scale rating						
		Negative ratings (1)	2	3	4	Positive ratings (5)	Average	SE
How confident are you in the following skills you learned during the pilot sessions?	Use knife skills in the kitchen	0	1	2	6	6	4.13	0.24
	Use basic cooking skills	0	0	1	9	1	4.00	0.13
	Cook from basic ingredients	0	1	0	6	8	4.40	0.21
	Follow a written recipe to prepare healthy food	0	0	1	5	9	4.53	0.17
	Did the pilot sessions' content meet your expectations?	1 (no)				14 (yes)	93.3%	
Session details	How would you rate the quality of the pilot sessions?	0	0	1	5	9	4.53	0.17
	Was the combination of PowerPoint presentations, discussion and activities suitable?	1 (no)				14 (yes)	93.3%	
	How was the pace of the sessions?	0 (slow)	4	9	1	1 (fast)	2.93	0.21
Reflection on session	Were the sessions useful to your needs as a future healthcare provider in counseling patients about the topic covered?	0	0	5	8	2	3.80	0.17
	How likely are you to take this elective if you are still at TUN by the time the elective is approved?	0	0	0	5	10	4.67	0.13
	How likely are you to recommend this elective to a classmate, a student from another cohort, or another program to take this elective?	0	0	1	2	12	4.73	0.15
	Is the list of topics to be covered by the elective course complete?	1 (no)				14 (yes)	93.3%	
	If you were to complete the elective based on the content indicated above, would it be enough to counsel, effectively, your future patients?	5 (no)				12 (yes)	58.3%	

## Discussion

This study explored the perception of undergraduate medical students regarding their preparedness to counsel patients and be a role model for a lifestyle which promotes positive health outcomes and prevents nutrition-related diseases. Since a large majority of the medical students surveyed communicated a desire for more nutrition education, an elective short experimental course was designed in the area of clinical nutrition complemented with culinary medicine as an active learning component. In response to student preferences, the experimental course was offered as an online PowerPoint, and the culinary workshops, which were in-person sessions, took place once every two weeks. Students reported gains in knowledge in nutrition education regarding the cardiovascular disease covered in the short experimental course, and improved skills associated with preparing adequate food for a person on a restricted salt and fat intake diet. They also expressed their willingness to participate in a longer elective, as well as recommending the elective to others. However, participants were less sure about how well the sessions would translate into clinical practice in the area of counseling patients.

The medical students in this study agreed with those in Hardman et al. [19] that (1) preventive health and nutrition education is part of the physicians’ job regardless of specialty, (2) nutrition counseling is an important part of patient interaction and can effectively influence patients to make healthy, effective lifestyle choices, and (3) that physicians are not adequately trained to advise patients in nutritional choices. Third- and fourth-year students were dissatisfied with the quality and quantity of their nutrition education, and wanted more time dedicated specifically to nutrition, as well as more integration of nutrition content into the new curriculum that is organ-system based and has no nutrition hours. The majority of third- and fourth-year students were unsure about their ability to counsel patients concerning their nutritional needs when nutrition is key to their health and well-being due to inadequate training in nutrition education. This finding corroborates a prior study where physicians recognized the need for more nutrition education in their training. Indeed, a study by Aggarwal et al. demonstrated that 58% of physicians had no memory of, or felt there was a notable absence of, nutrition education in their medical school training [20].

The proposed elective appears to be a promising strategy to inculcate not only the theoretical nutrition education to future physician, but also the practical skills required to effectively counsel patients on how to use food in their daily prevention or management of nutrition related diseases. There have been several other studies indicating that active learning is much more effective in imparting these skills to future physicians, hence the addition of culinary medicine as an active learning component of the proposed elective. Mauriello and Artz describe how culinary medicine lies at the intersection of cost-effective, healthy, and tasty foods [16]. Training health professionals, in a hands-on learning environment, can help support physicians to mentor patients on healthy eating and help reduce the burden of nutrition-related chronic diseases. Medical students at TUNCOM and those surveyed by Hardman et al. [19] agree that patients need specific instructions on how to eat a more nutritious and enjoyable diet; providing patients with specific directions to achieve a healthier diet, with reinforcement in subsequent visits can lead to long lasting behavioral changes. Placing students in the kitchen and learning how to prepare meals that are nutritious and palatable to themselves and their patients provide an active learning environment in hopes of giving future physicians confidence on how to effectively counsel patients on lifestyle choices. Furthermore, this gives physicians the tools so that they themselves can live healthier lifestyles. Spencer et al. have shown that physicians who practice healthy dietary and exercise habits are more likely to counsel patients on making these lifestyle changes [21].

**Fig. 4:**
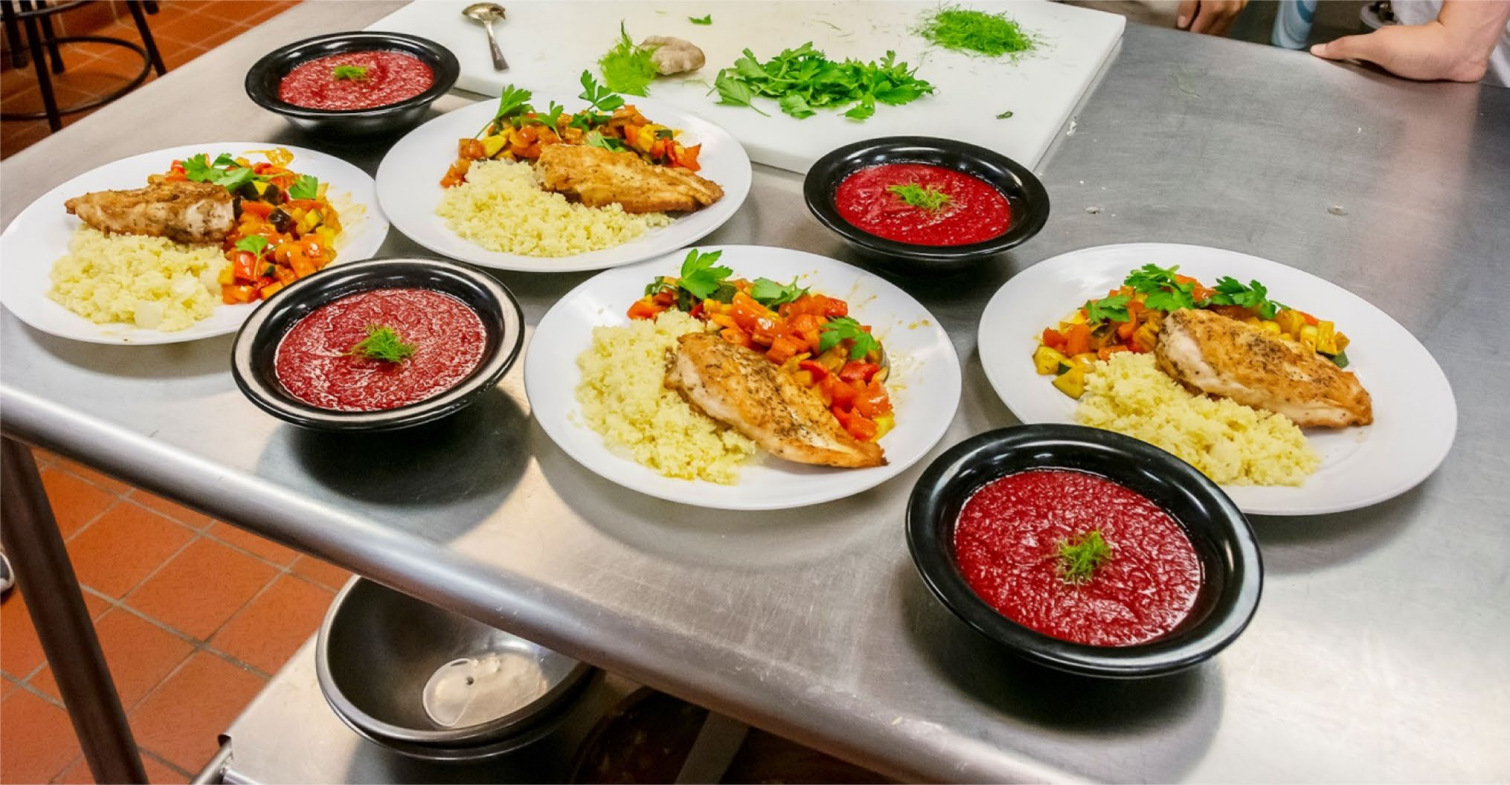
Meal prepared during the culinary medicine portion of the short experimental course

Although there is little room in the medical curriculum to add nutrition hours, there was strong demand from students for the nutrition elective. Stated preferences for a culinary medicine elective offered to medical students were used to design a short experimental course. The course was offered in an online format to help accommodate students who were doing rotations in their third and fourth years of schooling. The practical output from the culinary medicine portion was well-received (Fig. 4). The survey following the experimental course showed almost all participants in the short experimental session are likely to take the elective, if offered, and almost 80% of students deemed the sessions useful for their needs as future healthcare providers in counseling patients about coronary artery disease.

The student responses identified clinical application and usefulness for counseling future patients as the weakest area after the short experimental elective. The pre-workshop survey results suggest that students started out with low confidence that the elective would prepare them to counsel future patients. Given the initially low confidence, there are two likely reasons for low confidence in counseling patients. One is that the experimental elective did not include any specific instruction or practice in how to talk to patients about nutrition or other lifestyle changes. The other likely explanation is the brevity of the short experimental elective relative to the proposed full elective course. The entire elective would be comprised of eight didactic sessions and nine workshops of culinary medicine. Given the full elective and some instruction on counseling, it is expected that students’ confidence in counseling future patients would improve. An interesting follow-up to this study and something that is lacking in the current literature would be a survey of medical students who received the complete content of the clinical nutrition and culinary medicine elective so to assess the utility of the content in their practice as physicians.

### Limitations

Our study is not without limitations. Although we had very good response rates from first-year medical students, the second, third, and fourth-year student response rate was low. There is also a difference in curricula between the first-, second-, third- and fourth-year cohorts, although this did not seem to impact the data as there was still very little to no nutrition dedicated hours in the curriculum and responses were comparable among groups. Furthermore, students who signed up for the short experimental elective were self-selected, so there is an inherent selection bias, in both survey respondents and workshop participants, toward the students who may feel strongly about the role of nutrition in medical care.

## Conclusions

Our study identified an interest amongst TUNCOM students for an elective in clinical nutrition supplemented with culinary medicine workshops. The survey results showed medical students at TUNCOM wanted to have an elective held once every 2 weeks, with the didactic instructions provided through an online platform. Furthermore, third- and fourth-year students did not feel prepared, nor confident, in their abilities to counsel patients regarding nutritional goals. After the completion of the short experimental session, students showed a tremendous interest in the elective, and expressed that they would take a nutrition elective if it was offered.

A clinical nutrition elective with culinary medicine workshops would provide instruction for self-selected student doctors on how to advise patients with pertinent and specific instructions on how to adjust their lifestyle and eating habits to prevent or manage nutrition-based diseases.

## Supplementary Information

Below is the link to the electronic supplementary material.

Supplementary file1 (PDF 778 KB)

## Data Availability

Upon request.
