# Supplementary material for: A Suggested Strategy to Integrate an Elective on Clinical Nutrition with Culinary Medicine
Source: Med Sci Educ. 2021 Jul 6;31(5):1591–600. doi: 10.1007/s40670-021-01346-3 (PMC8446146; doi:10.1007/s40670-021-01346-3)
Supplement: Supplementary file 1 — Supplementary file1 (PDF 778 KB) [file 40670_2021_1346_MOESM1_ESM.pdf]

Table 1. Survey for 1st Year and 2nd Year Students

<u>Directions:</u> Each statement below finishes the sentence “In general, I believe that . . .” Select the response that is closest to your opinion about each statement.

1= Strongly Disagree, 5= Strongly Agree

1. Preventive health care is an important aspect of a Doctor of Osteopathic Medicine’s education.	1 2 3 4 5
2. As a Doctor of Osteopathic Medicine, preventive health care is an important aspect of any patient appointment.	1 2 3 4 5
3. Nutrition counseling should be part of routine care by all physicians, regardless of specialty.	1 2 3 4 5
4. Nutritional assessment and counseling should be included in any routine appointment, just like diagnosis and treatment.	1 2 3 4 5
*5. Nutrition counseling is ***not*** an effective use of my professional time.	1 2 3 4 5
6. Physicians have an impact on a patient’s ability to lose weight.	1 2 3 4 5
7. I have an obligation to improve the health of my patients including discussing nutrition with them.	1 2 3 4 5
8. All physicians, regardless of specialty, should counsel high-risk patients about dietary change.	1 2 3 4 5
9. It is worth my time to counsel patients with poor dietary patterns about nutrition.	1 2 3 4 5
10. Patients need healthy and yet good-tasting alternatives in order to change their eating patterns.	1 2 3 4 5
11. Most physicians are ***not*** adequately trained to discuss nutrition with patients.	1 2 3 4 5
12. Patients need specific instructions about how to change their eating behavior.	1 2 3 4 5
13. Specific advice about how to make dietary changes could help some patients improve their eating habits.	1 2 3 4 5
14. Patients need ongoing counseling following my initial instruction to maintain behavior changes consistent with a healthier diet.	1 2 3 4 5
15. Most patients will try to change their lifestyle if I advise them to do so.	1 2 3 4 5
16. Physicians can have an effect on a patient’s dietary behavior if they take the	1 2 3 4 5

time to discuss the problem.	
*17. For most patients, health education does ***little*** to promote adherence to a healthy lifestyle.	1 2 3 4 5
20. After receiving nutrition counseling, patients with poor eating habits will make major changes in their eating behavior.	1 2 3 4 5
21. After receiving nutrition counseling, patients with poor eating patterns will make moderate changes in their eating behavior.	1 2 3 4 5
22. My patient-education efforts will be effective in increasing patients' compliance with nutritional recommendations.	1 2 3 4 5

Please note that questions 5 and 17 are reverse scored and all questions except numbers 1 and 2 are derived from the Walsh et. al survey.

1. Will you be interested in joining an elective nutrition course with Culinary Medicine workshops to prepare you for your patients' counseling?
    a. Yes
    b. No
2. If you were interested in taking part in a nutrition elective, which method of instruction would you prefer?
    a. In class lectures and assessments, with Culinary Medicine workshops
    b. Online recorded lectures and assessments, required discussion posts and Culinary Medicine workshops
    c. Online PowerPoint based material and assessments, required discussion posts and Culinary Medicine workshops
3. Would you prefer the culinary medicine workshops be:
    a. Weekly
    b. Once every 2 weeks

The following questions relate to demographics:

1. What is your gender?
    a. Male
    b. Female
2. What is your age?
    a. 20-25
    b. 25-30
    c. 30-35
    d. 35-40
    e. 40-45
    f. 45-50
    g. >50

3. What is your ethnicity (optional)
    a. American Indian/Alaska Native
    b. Asian
    c. Black/African-American
    d. Native Hawaiian/Pacific Islander
    e. White
    f. Hispanic or Latino
    g. Unknown
    h. Other (please explain): ______________________
4. Did you have any nutrition training prior to medical school?
    a. No
    b. Yes, College coursework
    c. Yes, RD
    d. Yes, PhD
    e. Yes, Physical trainer
    f. Yes, Other (please explain): _______________________
5. Do you or any of your close family members or friends have any nutrition-linked medical problems that would require a greater than average knowledge of nutrition (i.e. Diabetes, BMI, etc.)?
    a. Yes
    b. No
6. Which of the following represents where your GPA stands thus far?
    a. <2.0
    b. 2.0-2.5
    c. 2.5-3.0
    d. 3.0-3.5
    e. 3.5-4.0
7. What is your intended specialty?
    a. Anesthesiology
    b. Dermatology
    c. Emergency Medicine
    d. Family Medicine
    e. Internal Medicine
    f. Neurology
    g. Obstetrics and Gynecology
    h. Ophthalmology
    i. Pathology
    j. Psychiatry
    k. Radiology
    l. Surgery
    m. Undecided

Table 2. Survey for 3rd Year and 4th Year Students

<u>DIRECTIONS:</u> The following questions measure your opinions about your nutrition education in medical school. Please indicate the extent to which you agree with the following statements.

1= Strongly Disagree, 5= Strongly Agree

1. I do ***not*** feel prepared to counsel patients regarding nutrition linked diseases	1 2 3 4 5
2. I am confident of my ability to counsel patients about nutrition.	1 2 3 4 5
3. I am ***not*** confident of my ability to counsel patients about nutrition.	1 2 3 4 5
4. My medical school curriculum had **little or no** nutrition education after the first year.	1 2 3 4 5
5. I am satisfied with the ***quantity*** of my nutrition education.	1 2 3 4 5
6. I am satisfied with the ***quality*** of my nutrition education.	1 2 3 4 5
7. My medical school nutrition curriculum should have had more time specifically dedicated to the topic of nutrition (independent of subject-based studies).	1 2 3 4 5
8. My medical school nutrition curriculum should have had more nutrition content formally integrated into the subject-based courses.	1 2 3 4 5
9. My medical school nutrition curriculum should have included more online materials available for independent study.	1 2 3 4 5
10. My medical school nutrition curriculum should have included more material relevant to my personal health and well-being.	1 2 3 4 5
11. My medical school nutrition curriculum should have been more scientifically rigorous.	1 2 3 4 5
12. My medical school curriculum **did not** have enough nutrition specific education.	1 2 3 4 5

Please note that questions 5-11 are derived from Walsh et. al survey and questions 4 and 12 are derived from Hardman et. al survey

1. Will you be interested in joining an elective nutrition course with Culinary Medicine workshops to prepare you for your patients' counseling?
   a. Yes
   b. No

2. If you were able to take part in a nutrition elective during third or fourth year, which method of instruction would you prefer.
    a. In class lectures and assessments, with Culinary Medicine workshops
    b. Online recorded lectures and assessments, required discussion posts and Culinary Medicine workshops
    c. Online PowerPoint based material and assessments, required discussion posts and Culinary Medicine workshops
3. Would you prefer the culinary medicine workshops be:
    c. Weekly
    d. Once every 2 weeks

The following questions relate to demographics:

1. What is your gender?
    a. Male
    b. Female
2. What is your age?
    a. 20-25
    b. 25-30
    c. 30-35
    d. 35-40
    e. 40-45
    f. 45-50
    g. >50
3. What is your ethnicity (optional)
    a. American Indian/Alaska Native
    b. Asian
    c. Black/African-American
    d. Native Hawaiian/Pacific Islander
    e. White
    f. Hispanic or Latino
    g. Unknown
    h. Other (please explain): ______________________
4. Did you have any nutrition training prior to medical school?
    a. No
    b. Yes, College coursework
    c. Yes, RD
    d. Yes, PhD
    e. Yes, Physical trainer
    f. Yes, Other (please explain): _______________________
5. Do you or any of your close family members or friends have any nutrition-linked medical problems that would require a greater than average knowledge of nutrition (i.e. Diabetes, IBM, etc.)?
    a. Yes
    b. No

6. Which of the following does your GPA lie in?
    a. <2.0
    b. 2.0-2.5
    c. 2.5-3.0
    d. 3.0-3.5
    e. 3.5-4.0
7. What is your intended specialty?
    a. Anesthesiology
    b. Dermatology
    c. Emergency Medicine
    d. Family Medicine
    e. Internal Medicine
    f. Neurology
    g. Obstetrics and Gynecology
    h. Ophthalmology
    i. Pathology
    j. Psychiatry
    k. Radiology
    l. Surgery
    m. Undecided

Table 3. Post-Workshop Survey

Thank you for accepting to complete this survey regarding the pilot sessions and the planned elective.

Please note that the content for the eight culinary workshops that are planned to be included in the elective of Clinical Nutrition with Culinary Medicine Sessions are as follows:

1. Knife skills, cutting, and chopping
2. Healthy meals for prevention and management for cardiovascular diseases
3. Healthy meals for prevention and management of Diabetes type 2 and metabolic syndrome
4. Healthy meals for people with food allergies
5. Nutritional vegetarian and vegan meals
6. Healthy eating on a limited budget
7. Food preparation that children will eat
8. Healthy desserts and healthy snacks

During the pilot sessions for Culinary Medicine as a component of the elective of Clinical Nutrition proposal, you have covered the first two sessions indicated above. You have also received a PowerPoint and a clinical case-based assessment with essay questions.

The completion of the eight sessions will result in a certificate and will be included in students' academic records.

Assuming that the elective included the above eight sessions with corresponding didactic material similar to the pilot via an online portal, please answer the following questions regarding the pilot sessions and the possible elective.

Please remember that the Culinary Medicine sessions will be scheduled from 6 to 8 PM (not including dinner time) every two weeks.

This survey will be available until January 12, 2020, and will be closed at 11:59 PM (PST).

Please complete it by this deadline.

<u>Pilot Sessions</u>

1. How confident are you in the following skills you learned during the pilot sessions?

a. Use knife skills in the kitchen
- Not confident
- Not very confident
- Neither confident nor unconfident
- Confident
- Very confident

b. Use basic cooking techniques
- Not confident
- Not very confident
- Neither confident nor unconfident
- Confident
- Very confident

c. Cook from basic ingredients (ex: fresh onions, carrots, raw chicken).
- Not confident
- Not very confident
- Neither confident nor unconfident
- Confident
- Very confident

d. Follow a written recipe to prepare healthy food (ex: preparing vegetable stock, grilled chicken, making a soup or a salad from fresh ingredients).
- Not confident
- Not very confident
- Neither confident nor unconfident
- Confident
- Very confident

2. Did the pilot sessions' content meet your expectations?

Yes

No, if this is your answer, please provide suggestions

3. How would you rate the quality of the pilot sessions?
Excellent
Good
Average
Poor
Terrible

4. Was the combination of PowerPoint presentations, discussion, and activities suitable?
Yes
No, if this is your answer, please provide suggestions

5. How was the pace of the pilot sessions?
Extremely fast
Somewhat fast
Average
Somewhat slow
Extremely slow

6. Were the sessions useful to your needs as a future healthcare provider in counseling patients about the topic covered?
Extremely useful
Very useful
Moderately useful
Slightly useful
Not at all useful

The following questions address the proposal for Clinical Nutrition with Culinary Medicine elective.

7. How likely are you to take this elective if you are still at TUN by the time the elective is approved?

Extremely likely
Somewhat likely
Neither likely nor unlikely
Somewhat unlikely
Extremely unlikely

8. How likely are you to recommend this elective to a classmate, a student from another cohort, or another program to take this elective?

Extremely likely
Somewhat likely
Neither likely nor unlikely
Somewhat unlikely
Extremely unlikely

9. Is the list of topics to be cover by the elective course complete (please see the list in the introductory message)?
   - Yes
   - No, If no, please make suggestions

10. If you were to complete the elective based on the content indicated above, would it be enough to counsel, effectively, your future patients?
    - Yes
    - No, if no, what else would you suggest?
